# A young male with shortness of breath

**DOI:** 10.4103/1817-1737.38001

**Published:** 2008

**Authors:** Fahmi Yousef Khan, Ahmed Al Ani, Mustafa S. Allaithy, Issam A Al-Bozom

**Affiliations:** *Department of Medicine, Hamad General Hospital, Doha, Qatar*

**Keywords:** Extragonadal seminoma, mediastinal seminoma

## Abstract

We report a case of primary mediastinal seminoma, which presented initially with shortness of breath and a swelling in upper part of anterior chest wall. The diagnosis of primary mediastinal seminoma was established on the basis of histologic findings and was confirmed by immunohistochemical analysis. Abdominal, pelvis and cerebral CT scan, testicular ultrasound and TC-99 MDP bone scintigraphy were negative. Chemotherapy was initiated with B.E.P. protocol (Bleomycin, Etoposide, Cisplatinum); the patient received four cycles of chemotherapy. After 8 months, the patient was seen in the clinic; he was well.

## Introduction

Primary seminomas of the mediastinum are unusual neoplasms that are morphologically indistinguishable from their gonadal counterparts but may have different biologic behavior because they arise at this particular location. The anterior mediastinum is the most common primary extragonadal site for seminoma. There are very few published reports of mediastinal seminomas located in middle and posterior mediastinum.

The case presented in this report highlights the importance of considering primary mediastinal seminoma in the differential diagnosis of anterior, middle and posterior mediastinal mass even if no abnormality of the testicle is observed.

## Case Report

A 32-year-old male patient was admitted to the hospital with a 6-month history of retrosternal chest pain which was described as compressive in character, localized, associated with mild shortness of breath and painful swelling in upper part of anterior chest wall. On direct questioning, he mentioned weight loss. Other medical history was unremarkable. On examination, the only abnormal physical sign was the presence of fixed tender swelling over manubrium sterni (2 × 3 cm).

**Figure 1:**
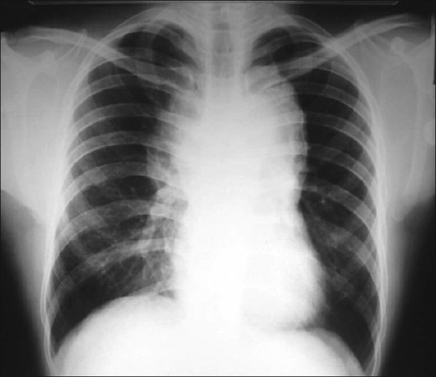
Plain chest X-ray shows large mediastinal mass

**Figure 2:**
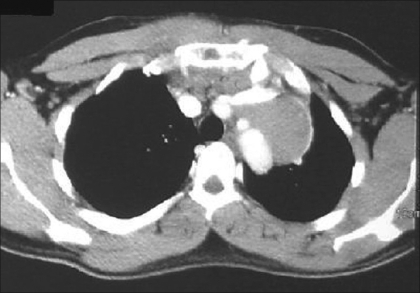
C-T Chest shows anterior superior mediastinal mass

Initial investigations revealed WBC 6,400/μL, hemoglobin 13.2 g/dl, platelets 324,000/μL, erythrocyte sedimentation rate 66 mm/hour. Blood chemistry, liver profile, arterial blood gas (ABG) analysis at room air and coagulations studies were within normal limits. A tuberculin skin test was negative. Chest X ray showed large mediastinal mass [Figure 1] and CT chest showed anterior superior mediastinal mass [Figure 2].

**Figure 3:**
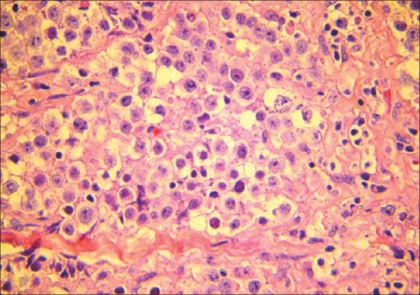
Lobules of large tumor cells, note the very prominent nucleoli and mature lymphocytes. (Hematoxylin and Eosin, ×400)

Fine needle aspiration of mediastinal mass was done and cytological diagnosis was noninformative. Bone marrow aspiration and trephine biopsy showed normal study. CT-guided biopsy was done, but unfortunately the specimen was crushed and full with artifacts. Open biopsy for mediastinal mass done by cardiothoracic surgeon and histological examination of the tissue obtained showed a malignant tumor that had two cell populations; one consisted of lobules, sheets, trabeculae and clusters of large polygonal cells with nuclei having vesicular chromatin, prominent nucleoli and several mitotic figures. The cytoplasm of those cells was moderately abundant and clear. The other cell population was clusters of small mature lymphocytes, which were scattered within the fibrous septa separating the lobules of the large polygonal tumor cells [Figure 3]. Immunoperoxidase stains in the polygonal tumor cells were positive for placental alkaline phosphates (PLAP) but negative for alpha fetoprotein, β -HCG, cytokeratin cocktail, Vimentin, S-100 protein, leukocyte common antigen (LCA), CD30 and CD15 (Hodgkin disease markers), CD20 (B-cell marker) and UCHL-1 (T-cell marker). Thus, diagnosis of mediastinal seminoma was established on the basis of histologic findings and was confirmed by immunohistochemical analysis.

Abdominal, pelvis and cerebral CT scan; testicular ultrasound and TC-99 MDP bone scintigraphy were negative. Tumor markers: α fetoprotein was 2.3 IU/ml (normal) and β-HCG was < 5 IU/L (normal).

Chemotherapy was initiated with B.E.P. protocol (Bleomycin, Etoposide, Cisplatinum); the patient received four cycles of the drugs. Two weeks after the first cycle, tumor size was reduced markedly. After 8 months, the patient was seen in the clinic; he was well.

## Discussion

Germ cell tumors occur most commonly in gonads but infrequently appear in other locations such as mediastinum, retroperitoneum, pineal gland and sacral area; 2-5% of germ cell tumors are of extragonadal origin.[1] It has been speculated that they occur in such unusual locations due to the abnormal migration of germ cells during embryogenesis.[2,3]

The primary mediastinal seminoma has an estimated incidence of 25% of that of primary mediastinal germ cell tumors and is usually located in the anterior mediastinum,[4,5] with the majority of cases occurring in males in their third decade.A MEDLINE search showed that only two cases of primary seminoma originating from the middle mediastinum[6,7] and two cases from the posterior mediastinum[8,9] have been reported in the literature. Our patient was 32 years old and the tumor was located in the anterior superior mediastinum.

The origin of extragonadal germ cell tumors (EGCTs) is still uncertain. The cytogenetic findings on chromosome 12q appear to suggest that extragonadal tumors would be of gonadal origin.[10]

Primary seminomas of the mediastinum are unusual neoplasms that are morphologically indistinguishable from their gonadal counterparts but may have different biologic behavior because they arise at this particular location.[11]

Histopathologically, germ cell tumors are classified as follows: teratomas (including mature and immature groups), germinomas (seminomas), embryonal carcinoma, choriocarcinoma, yolk sac tumors and mixed germ cell tumors.[12] Although they have similar histologic features, mediastinal GCTs are clinically and biologically distinct from their testicular counterparts.

Extragonadal germ cell tumors grow slowly and initially produce few symptoms (e.g., chest pain, dyspnea, cough). Around 30% of patients with mediastinal seminoma were asymptomatic and the mass was discovered incidentally during routine chest X ray.[11]

According to the results from an international analysis with 635 cases with extragonadal germ cell tumors, 83% (524) were nonseminomatous and 16% (104) seminomatous germ cell tumors.[13,14]

In this patient the differential diagnosis of mediastinal seminoma includes teratoma, primary large B cell lymphoma and thymoma. Some features commonly associated with seminoma, such as epitheloid granuloma, cystic changes and lymphocytic infiltration, were not prominent, but the application of immunohistochemical studies showed cytoplasmic staining with placental-like alkaline phosphatase. The application of immunohistochemical studies for placental-like alkaline phosphatase may be of discriminatory value in equivocal case.[11]

It is accepted that routine biopsy or orchidectomy is not needed in EGCTs if no evidence exists for retroperitoneal involvement and no abnormality of the testicle is observed.[4,15]

In our patient a careful clinical and ultrasound examination showed normal testis. Abdominal and pelvis CT showed no evidence of retroperitoneal involvement while brain CT scan was normal.

With regard to the treatment of mediastinal malignant GCTs, the current consensus is that initial systemic chemotherapy should be followed by aggressive complete resection of all macroscopic residual tumor when necessary.[16,17]

Prognosis for patients with mediastinal seminoma is fairly good, with a 5-year survival rate of around 90%. Conversely, nonseminomas have a worse prognosis than seminoma, with a 5-year survival rate of around 45%.[13,14] Patients with extragonadal germ cell tumors are treated at the present time with three or four cycles of Cisplatinum, Etoposide, Bleomycin.

Thus, mediastinal seminoma should be considered in the differential diagnosis of middle, posterior and anterior superior mediastinal mass even in the absence of testicle lesions.
